# Oral Typhoid Vaccination With Live-Attenuated *Salmonella* Typhi Strain Ty21a Generates Ty21a-Responsive and Heterologous Influenza Virus–Responsive CD4^+^ and CD8^+^ T Cells at the Human Intestinal Mucosa

**DOI:** 10.1093/infdis/jiw030

**Published:** 2016-01-24

**Authors:** Shaun H. Pennington, Ameeka L. Thompson, Adam K. A. Wright, Daniela M. Ferreira, Kondwani C. Jambo, Angela D. Wright, Brian Faragher, Jill W. Gilmour, Stephen B. Gordon, Melita A. Gordon

**Affiliations:** 1 Department of Clinical Infection, Microbiology, and Immunology, Institute of Infection and Global Health, University of Liverpool; 2 Department of Clinical Sciences, Liverpool School of Tropical Medicine; 3 International AIDS Vaccine Initiative, Human Immunology Laboratory, Imperial College, London, United Kingdom

**Keywords:** *Salmonella*, typhoid, Ty21a, cellular immunity, T cells, cytokines, humoral immunity, immunoglobulins, heterologous immunity

## Abstract

***Background.*** Oral vaccination with live-attenuated *Salmonella* Typhi strain Ty21a is modestly efficacious, but the mechanisms of protection are currently unknown. While humoral and cellular immune responses are well described in peripheral blood, the cellular response at the intestinal mucosa has never been directly assessed.

***Methods.*** We vaccinated healthy adults with Ty21a and assessed humoral and cellular immunity in vaccinated volunteers and controls after 18 days. Immunoglobulin levels were assessed in peripheral blood by an enzyme-linked immunosorbent assay. Cellular responses were assessed in peripheral blood and at the duodenal and colonic mucosa by flow cytometry.

***Results.*** We demonstrate the generation of Ty21a-responsive and heterologous influenza virus–responsive CD4^+^ and CD8^+^ T cells at the duodenal mucosa. All duodenal responses were consistently correlated, and no responses were observed at the colonic mucosa. Peripheral anti-lipopolysaccharide immunoglobulin G and immunoglobulin A responses were significantly correlated with duodenal responses. The assessment of integrin β_7_ expression intensity among peripheral and duodenal T-cell subsets revealed varied capacities for mucosal homing and residence.

***Conclusions.*** The breadth of duodenal cellular responses was not reflected peripherally. The direct evaluation of mucosal immune defense may yield functional correlates of protection and could provide insight into mechanisms that may be manipulated to enhance vaccine immunogenicity.

*Salmonella enterica* serovar Typhi is a human-host-restricted intracellular pathogen and the causative agent of typhoid fever. Following ingestion, the bacteria cause systemic illness following invasion via the mucosal surface of the small intestine [1]. In the 1970s, through chemical mutagenesis of pathogenic *S*. Typhi strain Ty2, a live-attenuated oral typhoid vaccine, Ty21a, was developed [2]. Vaccination with 3 doses of Ty21a is moderately protective, and although estimates of efficacy vary [3–5], a recently published review calculated a cumulative efficacy of 48% 3 years following vaccination [6].

Ty21a is able to induce humoral and cellular immune responses, both of which have been implicated in protection against disease. While opsonophagocytic antibody function [7], cellular cytotoxicity, proliferation, and cytokine production functionality have been assessed in peripheral blood following vaccination with Ty21a [8–13], cellular immunity at the human intestinal mucosa has never been directly assessed. Numerous studies have demonstrated that cellular immune responses generated through vaccination with Ty21a are primed for mucosal homing [13–15], highlighting the importance of mucosal immunity in defense against disease. Furthermore, it has been demonstrated that the assessment of vaccine immunogenicity by peripheral sampling alone provides an incomplete reflection of vaccine immunogenicity [16].

It has previously been observed in murine models that previously primed T cells of heterologous specificities are recruited to the lung during influenza virus infection [17]. Although this phenomenon has not been observed in humans, we hypothesized that vaccination with Ty21a could enhance T-cell responses to heterologous antigens at the mucosal surface by a similar mechanism.

Through the direct assessment of immunity at the intestinal mucosa, it may be possible to identify mechanisms involved in the induction of protective immunity, which may be manipulated to improve oral vaccine immunogenicity. Here, we have assessed cellular immunity in vaccinated volunteers and controls at the duodenal and colonic mucosa and in peripheral blood after 18 days. We have compared and correlated peripheral and mucosal cellular responses with accepted peripheral humoral measures of vaccine efficacy, providing a unique insight into the relationship between human mucosal and peripheral immune defense.

## MATERIALS AND METHODS

### Ethical Approval, Recruitment, and Study Protocol

All volunteers provided written informed consent. This study was approved by the United Kingdom National Research Ethics Service (10/H1005/20). Twenty-three healthy adult volunteers were enrolled into the study. Eleven volunteers (6 males and 5 females [2 of whom were previously vaccinated against influenza]; median age, 24 years) were randomly selected for oral vaccination with live-attenuated *S*. Typhi (Ty21a; Vivotif). A single oral capsule was taken on days 0, 2, and 4, approximately 1 hour before a meal, with a cold or lukewarm drink. Twelve volunteers (3 males and 9 females [3 of whom were previously vaccinated against influenza]; median age, 23 years) were randomly assigned to an unvaccinated control group.

### Mucosal Mononuclear Cell (MMC) Isolation

Mucosal samples were acquired at day 18. O_2_ was administered nasally, and saturation was monitored throughout endoscopic biopsy. Sedation was offered to all volunteers; those who requested sedation were given up to 5 mg of midazolam intravenously. By use of large-capacity forceps (Boston Scientific), 12–15 single-bite mucosal biopsy specimens were acquired during flexible video-endoscopy from the duodenal mucosa at parts D2–D3 (n = 20) and from the sigmoid colon at an insertion distance of 20–25 cm (n = 17). MMCs were isolated from biopsy specimens, using a modified version of a previously described method [31]. Full details are presented in the Supplementary Materials and Methods.

### Peripheral Blood Mononuclear Cell (PBMC) Isolation

Peripheral blood samples were collected in sodium heparin Vacutainers (BD Biosciences) at day 0 (n = 23) and, after overnight fasting, at day 18 (n = 21). PBMCs were isolated using Lymphoprep (Axis-Shield), according to the manufacturers instructions. Full details are presented in the Supplementary Materials and Methods.

### Antigenic Stimulation and Incubation

PBMCs (1 × 10^6^ cells/well) and MMCs (between 0.5 × 10^6^ and 1 × 10^6^ cells/well) were seeded in complete medium in 96-well flat-bottomed plates. Cells in each well were stimulated with either 1 × 10^6^ colony-forming units (CFU) heat-killed *Salmonella* Typhi Ty21a (Vivotif; bacteria were suspended in Dulbecco's phosphate-buffered saline [PBS], quantified using the Miles and Misra technique, and killed by incubation at 95°C for 30 minutes) or 45 ng influenza virus hemagglutinin and neuraminidase antigens (Influvac, containing, in equal quantities, a A/Brisbane/59/2007 H_1_N_1_-like strain, a A/Brisbane/10/2007 H_3_N_2_-like strain, and a B/Brisbane/60/2008-like strain). One positive control well was stimulated with 100 ng staphylococcal enterotoxin B (SEB; Sigma-Aldrich). One negative control well was left untreated to adjust for non–antigen-specific background cytokine production. Cells were then incubated at 37°C in 5% CO_2_. After 2 hours, 1 µL brefeldin A (BD GolgiPlug; BD Biosciences) was added to each well, and the plate was incubated for a further 16 hours at 37°C in 5% CO_2_.

### Flow Cytometric Analyses

Following incubation, PBMCs and MMCs were washed, stained for viability and surface phenotype, and, following fixation and permeabilization, stained for intracellular cytokine production. Details of the antibodies that were used are presented in the Supplementary Materials and Methods. Cells were washed, resuspended, and stored in the absence of light at 4°C until data were acquired using a LSR II flow cytometer (BD Biosciences). Compensation beads (BD Biosciences) were used to create compensation matrices, and sequential cell isolation was used to identify populations of interest (Figure 2). Full details are presented in the Supplementary Materials and Methods.

### Enzyme-Linked Immunosorbent Assay (ELISA)

Each well in flat-bottomed 96-well microtitre plates (Nunc) was coated with 100 µL of carbonate-bicarbonate buffer containing either 50 ng *S*. Typhi lipopolysaccharide (LPS; Sigma-Aldrich) or 25 ng influenza virus hemagglutinin and neuraminidase antigens (Influvac, containing, in equal quantities, a A/Brisbane/59/2007 H_1_N_1_-like strain, a A/Brisbane/10/2007 H_3_N_2_-like strain, and a B/Brisbane/60/2008-like strain) and incubated at 4°C overnight. Plates were washed 3 times with PBS-Tween. Plates were blocked with 1.0% bovine serum albumin and incubated for 2 hours at room temperature. A standard was created using serum obtained from a convalescent patient with a diagnosis of typhoid. Volunteer samples were diluted 4 times across an optimized range for optimum comparison against the standard. Plates were washed, and samples were added in duplicate and incubated at 4°C overnight. For detection of immunoglobulin G (IgG), plates were washed and incubated with 1:4000 anti-human IgG–alkaline phosphatase (Sigma-Aldrich) for 2 hours. For detection of immunoglobulin A (IgA), plates were washed and incubated with 1:4000 anti-human-IgA (AbD Serotec) for 2 hours; plates were washed again and then incubated with 1:2000 streptavidin to alkaline phosphatase (AbD Serotec) for 1 hour. For detection of both IgG and IgA, plates were washed and incubated with 100 µL *p*-nitrophenyl phosphate (Sigma-Aldrich). Optical density was measured at 405 nm, using a FLUOstar Omega microplate reader (BMG Labtech).

### Statistical Analyses

Comparisons were made using paired and unpaired *t* tests, as indicated. Associations were measured using the Pearson correlation coefficient. Statistical analyses were performed using Prism v5.03 (GraphPad). *P* values are 2-tailed and considered significant at *P* < .05.

## RESULTS

### Serum Immunoglobulin Specificity

Ty21a-mediated protection is dependent upon the expression of LPS [2], and, in field trials, humoral responses to LPS were shown to correlate with vaccine efficacy [18]. We compared levels of serum anti-LPS IgG and IgA prior to and following vaccination. We also measured levels of serum IgG and IgA specific to influenza virus, a common naturally encountered pathogen, to assess the impact of vaccination on humoral immunity to a heterologous pathogen. Influenza virus was selected since the majority, if not all, volunteers would have been exposed to this pathogen in the community.

**Figure 1.**
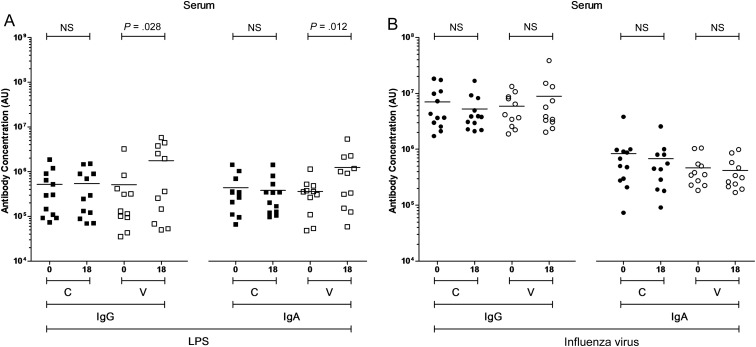
Levels of serum immunoglobulin G (IgG) and immunoglobulin A (IgA) to specific antigens. The levels of IgG and IgA specific to *Salmonella* Typhi lipopolysaccharide (LPS; *A*) and influenza virus (*B*) in serum, expressed in arbitrary units (AU). For control (C; closed squares and circles) and vaccinated (V; open squares and circles) volunteers, paired comparisons were made between day 0 and day 18 values. Horizontal bars represent mean values (paired *t* tests were performed using logarithmically transformed data). Abbreviation: NS, not significant.

While levels of anti-LPS serum IgG and IgA among unvaccinated subjects were not different between day 18 and day 0, levels among the vaccinated were higher at day 18 than at day 0 (*P* = .03 and *P* = .01; Figure 1*A*). Levels of anti-influenza serum IgG or IgA among the unvaccinated and the vaccinated subjects were not different between day 18 and day 0 (Figure 1*B*).

### Peripheral Blood and Gut Mucosal Cellular Responses

**Figure 2.**
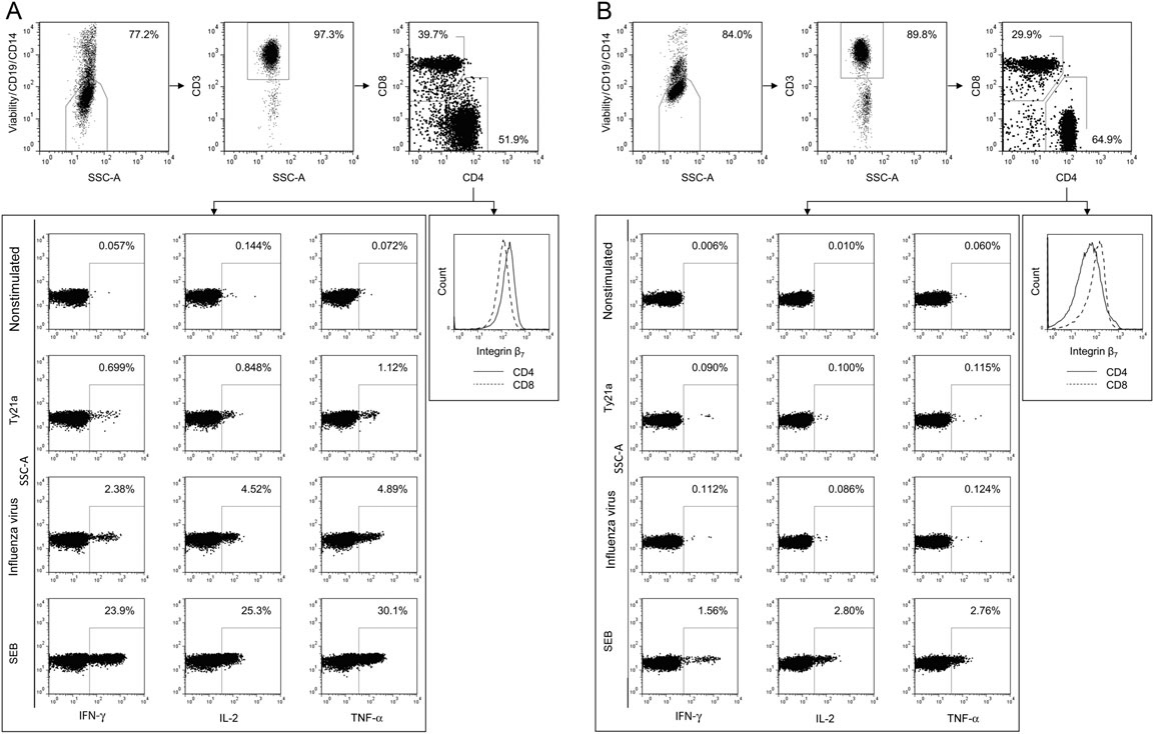
Representative flow cytometric gating strategy. Dot plots are shown for cells isolated from the duodenal mucosa (*A*) and peripheral blood (*B*). Dead cells, B cells, and monocytes were removed by staining for viability (Vivid), CD19, and CD14 and gating on the negative population. T cells were identified according to the expression of CD3. T cells were classified according to the expression of CD4 and CD8 and the expression of interferon γ (IFN-γ), tumor necrosis factor α (TNF-α), and/or interleukin 2 (IL-2) assessed in nonstimulated and in live-attenuated *Salmonella* Typhi strain Ty21a (Ty21a)-, influenza virus–, and staphylococcal enterotoxin B (SEB)–stimulated samples.

We compared the frequency of Ty21a-responsive T cells in vaccinated volunteers and controls, at the duodenal and colonic mucosa and in peripheral blood. We also measured the frequency of influenza virus–responsive T cells. A combinatorial gating strategy was used to identify the proportion of CD4^+^ and CD8^+^ T cells positive for any combination of interferon γ (IFN-γ), tumor necrosis factor α (TNF-α), and/or interleukin 2 (IL-2; Figure 2). Cytokine production in nonstimulated samples (negative control) was minimal, did not differ between vaccinated and unvaccinated subjects, and was subsequently subtracted from other conditions. Cytokine production in staphylococcal enterotoxin B–stimulated samples (positive control) was high and did not differ between vaccinated and unvaccinated subjects.

At day 0, in peripheral blood, the frequency of Ty21a-responsive and heterologous influenza virus–responsive CD4^+^ and CD8^+^ T cells in the vaccinated group was not different from the frequency in the unvaccinated control group (Supplementary Figure 1). These data suggest that groups were well matched for prior exposure to Ty21a and influenza virus antigens and that any differences observed thereafter may be attributed to an effect of vaccination with Ty21a. Paired comparisons between day 0 and day 18 were not made in peripheral blood, as overnight fasting, required prior to endoscopy, is known to influence cytokine production in response to restimulation with bacterial and viral antigens [19].

At day 18, at the duodenal mucosa, the frequency of Ty21a-responsive CD4^+^ T cells was 3-fold higher and the frequency of Ty21a-responsive CD8^+^ T cells 5-fold higher in vaccinated volunteers, compared with unvaccinated volunteers (*P* = .007 and *P* = <.0001, respectively; Figure 3*A*). The frequency of heterologous influenza virus–responsive CD4^+^ T cells was 5-fold higher and the frequency of heterologous influenza virus–responsive CD8^+^ T cells 6-fold higher in vaccinated volunteers, compared with unvaccinated volunteers (*P* = .005 and *P* = .01, respectively; Figure 3*A*). At the colonic mucosa, there was no significant difference between the frequencies of Ty21a-responsive or heterologous influenza virus–responsive T cells in vaccinated volunteers, compared with unvaccinated volunteers (Figure 3*B*).

**Figure 3.**
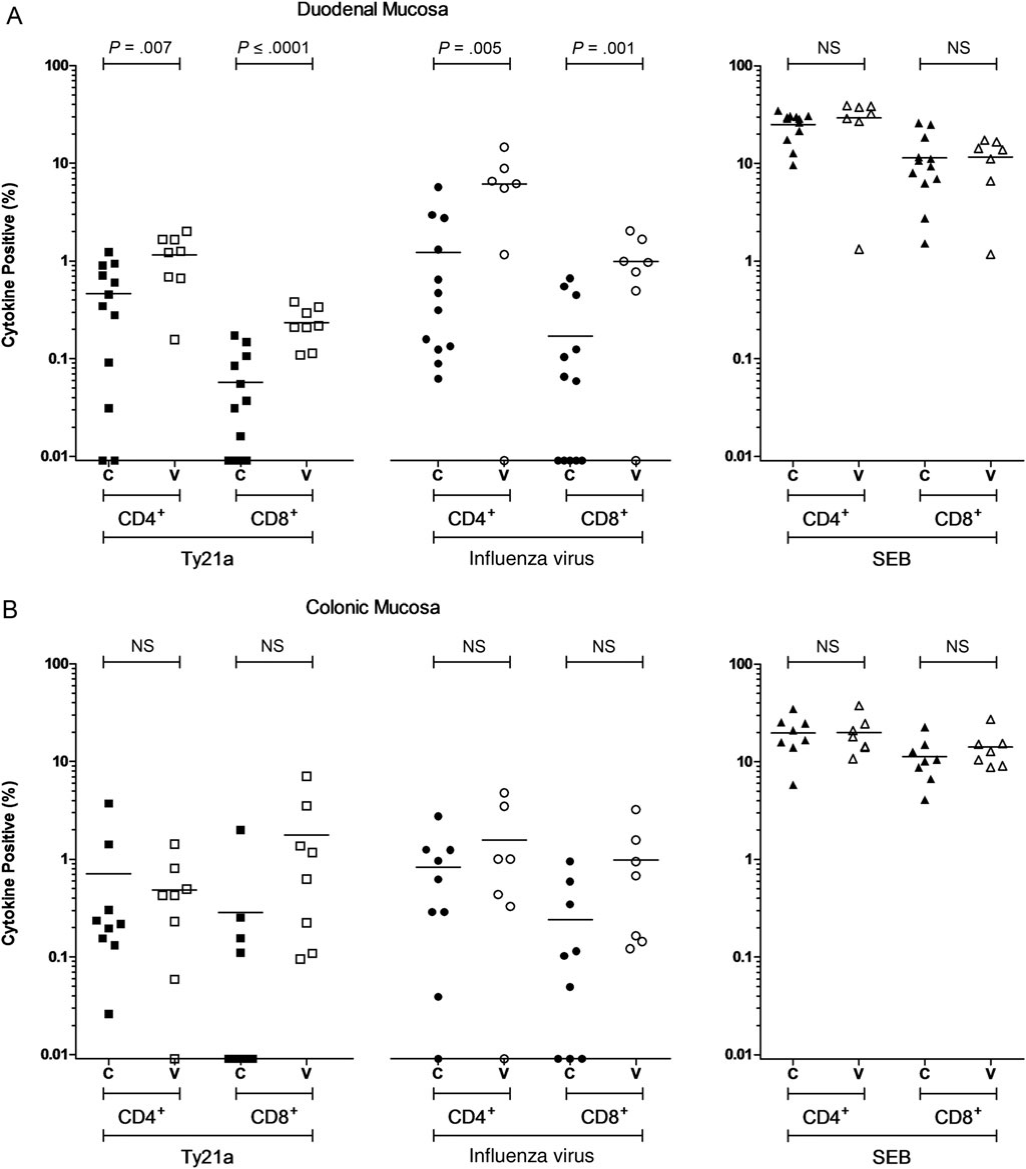
Antigen-specific cytokine-producing populations at day 18. The frequency of CD4^+^ and CD8^+^ live-attenuated *Salmonella* Typhi strain Ty21a (Ty21a)-responsive and heterologous influenza virus–responsive populations expressing any combination of interferon γ, tumor necrosis factor α, and/or interleukin 2 above background. Staphylococcal enterotoxin B (SEB)–stimulated control data is also included. For control (C; closed squares, circles, and triangles) and vaccinated (V; open squares, circles, and triangles) volunteers, measurements were made at the duodenal mucosa (*A*), the colonic mucosa (*B*), and in peripheral blood (*C*). Horizontal bars represent mean values (comparisons were made using unpaired *t* tests). Abbreviation: NS, not significant.

**Figure 3:**
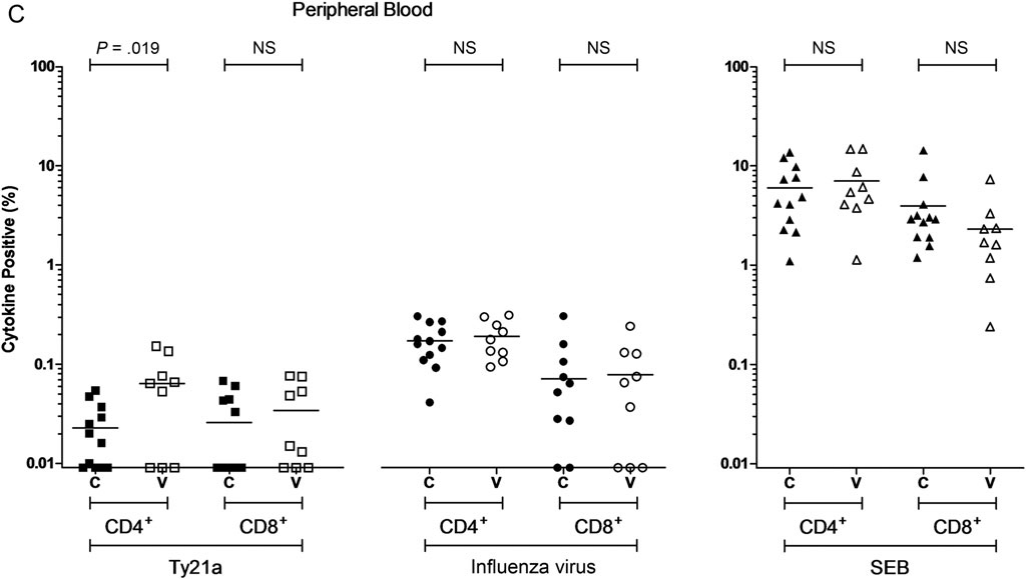

In peripheral blood, the frequency of Ty21a-responsive CD4^+^ T cells was 4-fold higher in vaccinated volunteers, compared with unvaccinated volunteers (*P* = .019; Figure 3*C*). Vaccination did not influence the frequency of peripheral Ty21a-responsive CD8^+^ T cells, nor the frequency of heterologous influenza virus–responsive CD4^+^ or CD8^+^ T cells at day 18.

### Characteristics and Functionality of Cellular Responses

Polyfunctional T cells, defined as cells that express multiple cytokines simultaneously, have been shown to correlate with vaccine-mediated protection against other intracellular infections [20, 21]. We compared the cytokine expression profile of vaccinated volunteers with that of unvaccinated volunteers to assess the functionality of responses to Ty21a and influenza virus antigens.

**Figure 4.**
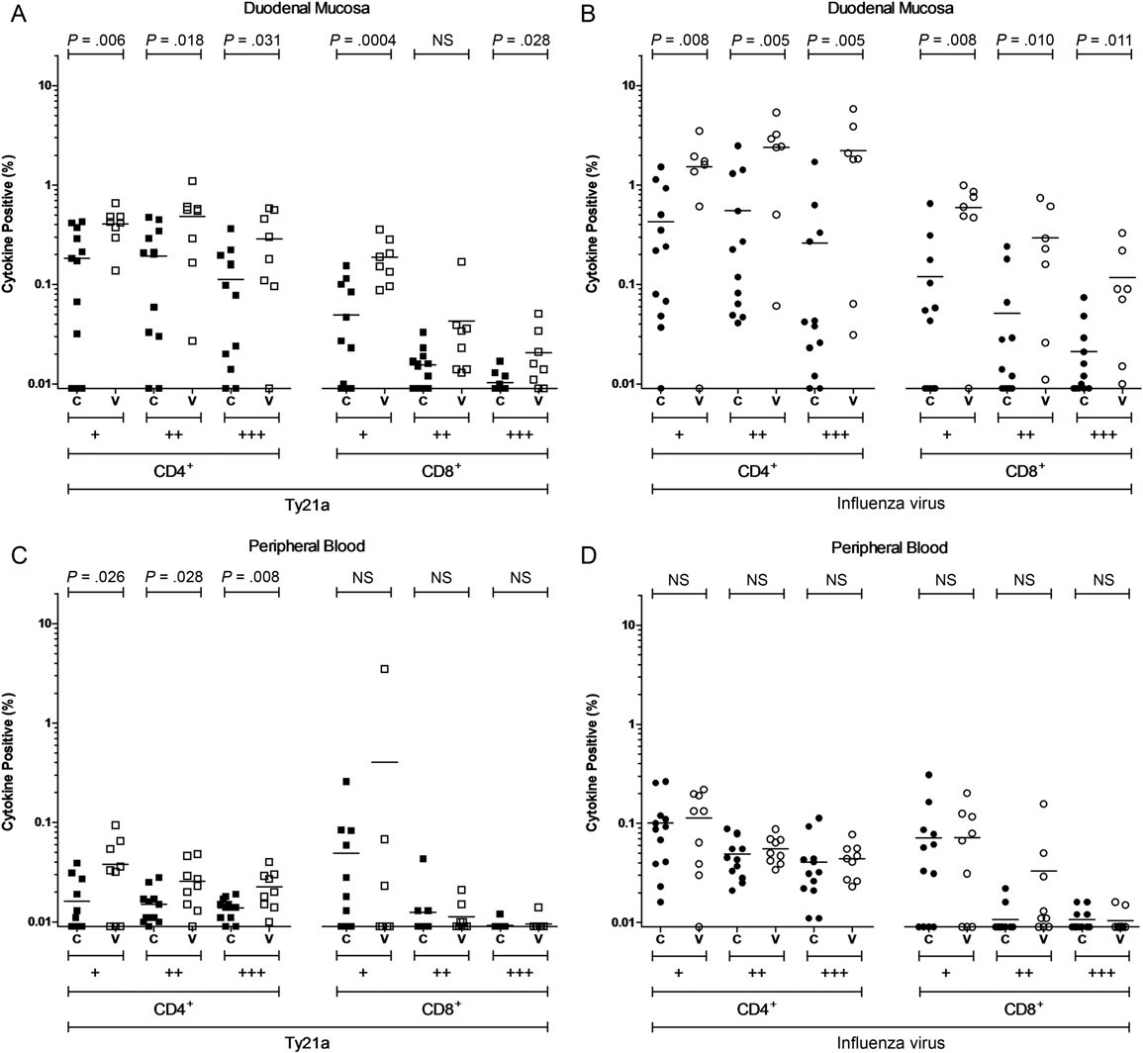
Combinations of antigen-specific cytokine production at day 18. The frequency of CD4^+^ and CD8^+^ live-attenuated *Salmonella* Typhi strain Ty21a (Ty21a)-responsive and heterologous influenza virus–responsive populations expressing 1 (+), 2 (++), or 3 (+++) cytokines (interferon γ, tumor necrosis factor α, and/or interleukin 2) above background. For control (C; closed squares, circles, and triangles) and vaccinated (V; open squares, circles, and triangles) volunteers, measurements were made at the duodenal mucosa (*A* and *B*) and in peripheral blood (*C* and *D*). Horizontal bars represent mean values (comparisons were made using unpaired *t* tests). Abbreviation: NS, not significant.

At the duodenal mucosa, the CD4^+^ T-cell response to Ty21a was functionally heterogeneous, with the frequency of cells expressing 1, 2, or 3 cytokines being higher in vaccinated volunteers, compared with unvaccinated volunteers (*P* = .006, *P* = .018, and *P* = .031, respectively; Figure 4*A*). The duodenal CD8^+^ T-cell response to Ty21a antigens was, in contrast, largely attributable to cells expressing just 1 cytokine (*P* = .0004; Figure 4*A*). Duodenal CD4^+^ and CD8^+^ T-cell responses to influenza virus antigens were also functionally heterogeneous, with the frequency of cells expressing 1, 2, or 3 cytokines being higher in vaccinated volunteers, compared with unvaccinated volunteers (CD4^+^ T cells, *P* = .008, *P* = .005, and *P* = .005, respectively; CD8^+^ T cells, *P* = .0008, *P* = .010, and *P* = .011; Figure 4*B*).

In peripheral blood, the CD4^+^ T-cell response to Ty21a antigens was functionally heterogeneous, with the frequency of cells expressing 1, 2, or 3 cytokines being higher in vaccinated volunteers, compared with unvaccinated volunteers (*P* = .026, *P* = .028, and *P* = .008, respectively; Figure 4*C*). Analysis of expression profiles by individual cytokine demonstrated that responses to Ty21a and influenza virus antigens were attributable to cells producing combinations of IFN-γ, TNF-α, and IL-2 but that no one cytokine predominated (Supplementary Figure 2).

### Correlations Between Cellular Populations at the Duodenal Mucosa

We explored the relationship between the generation of Ty21a-responsive and heterologous influenza virus–responsive T cells at the duodenal mucosa. The frequencies of Ty21a-responsive CD4^+^ and CD8^+^ T cells were correlated (r^2^ = 0.537 and *P* = .015; Table 1), as were the frequencies of heterologous influenza virus–responsive CD4^+^ and CD8^+^ T cells (r^2^ = 0.930 and *P* = <.0001; Table 1). The frequencies of Ty21a-responsive CD4^+^ and CD8^+^ T cells also correlated robustly with the frequencies of corresponding influenza virus–responsive T cells (CD4^+^ T cells, r^2^ = 0.74 and *P* = .0003; CD8^+^ T cells, r^2^ = 0.677 and *P* = .001; Table 1).

**Table 1.** Pearson Correlation Analysis of Cellular and Humoral Immune Responses

Variable	Duodenal Mucosa				Peripheral Blood				Serum			
	Ty21a		Influenza Virus		Ty21a		Influenza Virus		*S.* Typhi LPS		Influenza Virus	
	CD4^+^ T Cells	CD8^+^ T Cells	CD4^+^ T Cells	CD8^+^ T Cells	CD4^+^ T Cells	CD8^+^ T Cells	CD4^+^ T Cells	CD8^+^ T Cells	IgG	IgA	IgG	IgA
Duodenal mucosa												
Ty21a												
CD4^+^ T cells	1	0.537^a^	0.741^b^	0.724^b^	0.530^a^	0.319	−0.102	−0.223	0.542^a^	0.710^a^	0.160	0.008
CD8^+^ T cells	…	1	0.587^b^	0.677^b^	0.395	0.126	−0.117	−0.232	0.315	0.396	0.177	−0.348
Influenza virus												
CD4^+^ T cells	…	…	1	0.929^b^	0.327	0.299	−0.025	−0.122	0.392	0.512^a^	−0.245	−0.228
CD8^+^ T cells	…	…	…	1	0.394	0.515	0.099	0.080	0.392	0.585^b^	−0.149	−0.300

Unless otherwise indicated, values were not statistically significant.

Abbreviations: IgA, immunoglobulin A; IgG, immunoglobulin G; LPS, lipopolysaccharide; *S.* Typhi, *Salmonella enterica* serovar Typhi; Ty21a, live-attenuated *Salmonella* Typhi strain Ty21a.

^a^ *P* < .05.

^b^ *P* < .001.

### Correlations Between Peripheral and Mucosal Immunity

We explored the relationship between the generation of peripheral and duodenal immune responses. The frequency of peripheral Ty21a-responsive CD4^+^ T cells was modestly correlated with the frequency of duodenal Ty21a-responsive CD4^+^ T cells (r^2^ = 0.530 and *P* = .024; Table 1) but not with the frequency of duodenal Ty21a-responsive CD8^+^ T cells or with the frequency of heterologous influenza virus–responsive CD4^+^ and CD8^+^ T cells. Levels of serum anti-LPS IgG and IgA correlated with the frequency of duodenal Ty21a-responsive CD4^+^ T cells (IgG, r^2^ = 0.542 and *P* = .014; IgA, r^2^ = 0.710 and *P* = .0004; Table 1). Levels of serum IgA specific to LPS also correlated with the frequency of duodenal heterologous influenza virus–responsive CD4^+^ and CD8^+^ T cells (CD4^+^ T cells, r^2^ = 0.512 and *P* = .025; CD8^+^ T cells, r^2^ = 0.585 and *P* = .008; Table 1). Levels of anti-influenza virus IgG and IgA did not, however, correlate with either the frequency of Ty21a-responsive or heterologous influenza virus–responsive T cells at the duodenal mucosa in either subset.

### Cellular Potential for Mucosal Homing and Residence

Integrin β_7_ plays a prominent role in mucosal cellular immune defense: α_4_β_7_ facilitates peripheral trafficking to the intestinal mucosa, and α_ε_β_7_ helps maintain resident intraepithelial lymphocyte populations [22, 23]. Since duodenal CD4^+^ and CD8^+^ T-cell responses to Ty21a and influenza virus antigens were closely correlated, we assessed the cellular capacity for mucosal homing and residence, which is a potential mechanism by which Ty21a vaccination may have influenced trafficking to the duodenal mucosa. We assessed integrin β_7_ expression intensity, a component of both α_4_β_7_ in peripheral blood and α_ε_β_7_ at the duodenal mucosa. A combinatorial gating strategy was used to determine the level of integrin β_7_ expression among CD4^+^ and CD8^+^ T-cell populations (Figure 2).

**Figure 5.**
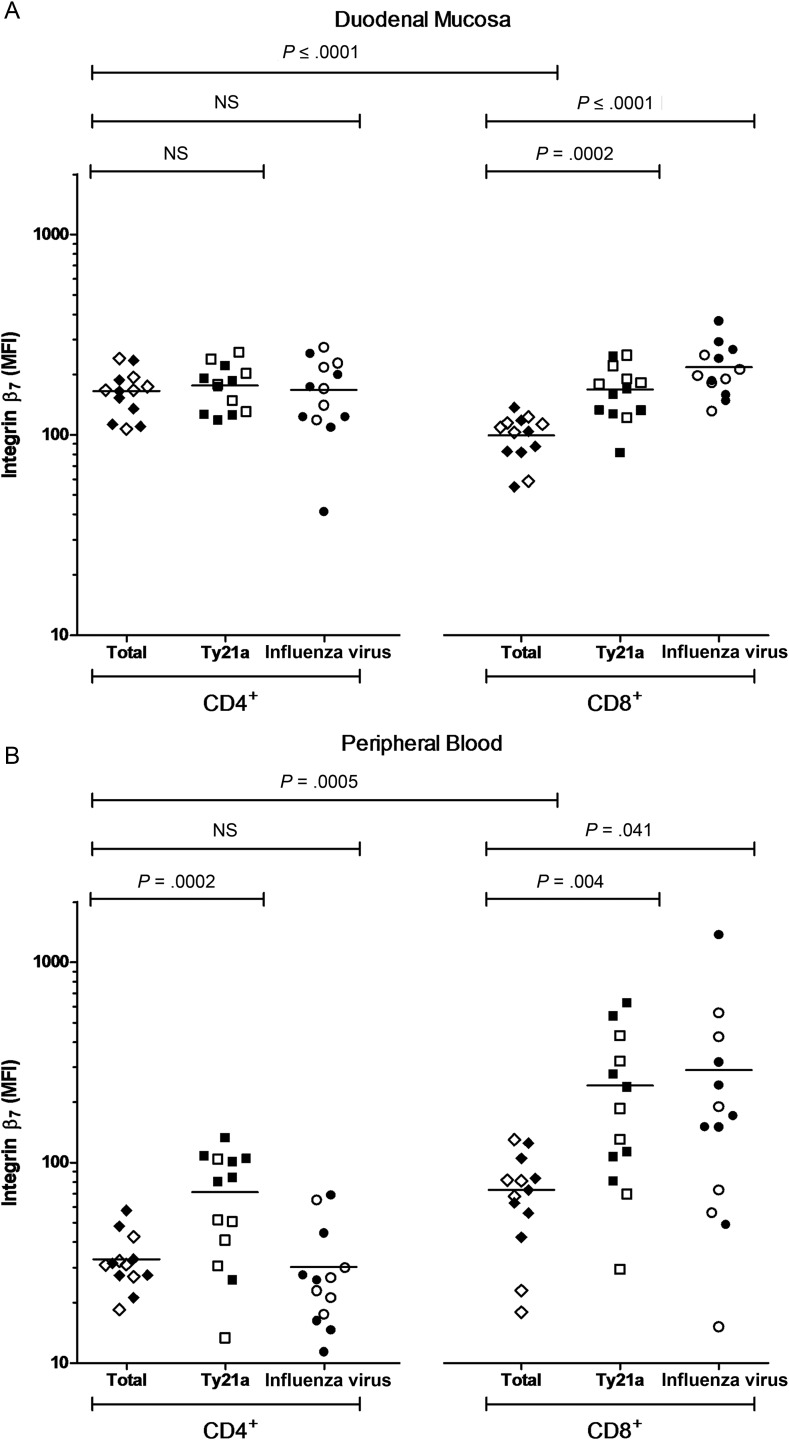
Integrin β_7_ expression intensity among T-cell populations at day 18. Geometric mean fluorescence intensity (MFI) expression of integrin β_7_ among nonstimulated total CD4^+^ and total CD8^+^ T-cell populations (control volunteers, closed diamonds; vaccinated volunteers, open diamonds) and in live-attenuated *Salmonella* Typhi strain Ty21a (Ty21a)-stimulated (control volunteers, closed squares; vaccinated volunteers, open squares) and heterologous influenza virus–stimulated (control volunteers, closed circles; vaccinated volunteers, open circles) cytokine-producing subpopulations. Measurements were made at the duodenal mucosa (*A*) and in peripheral blood (*B*). Horizontal bars represent mean values (comparisons were made using unpaired *t* tests). Abbreviation: NS, not significant.

At the duodenal mucosa, integrin β_7_ expression was more intense among the total CD4^+^ T-cell population than among the total CD8^+^ T-cell population (*P* = <.0001; Figure 5*A*). Integrin β_7_ expression intensity among Ty21a-responsive and influenza virus–responsive CD4^+^ T-cell subpopulations was not different from that among the total mucosal CD4^+^ T-cell population. In contrast, integrin β_7_ expression intensity among Ty21a-responsive and influenza virus–responsive CD8^+^ T-cell subpopulations was greater than that among the total mucosal CD8^+^ T-cell population (*P* = .0002 and *P* = <.0001, respectively; Figure 5*A*).

In contrast with observations made at the duodenal mucosa, integrin β_7_ expression was more intense among the total CD8^+^ T-cell population than among the total CD4^+^ T-cell population in peripheral blood (*P* = .0005; Figure 5*B*). While integrin β_7_ expression intensity among the Ty21a-responsive CD4^+^ T-cell subpopulation was greater than that among the total CD4^+^ T-cell population (*P* = .002; Figure 5*B*), expression intensity among the influenza virus–responsive CD4^+^ T-cell subpopulation was not different from that among the total CD4^+^ T-cell population. Integrin β_7_ expression intensity among the Ty21a-responsive and heterologous influenza virus–responsive CD8^+^ T-cell subpopulations was greater than that among the total CD8^+^ T-cell population (*P* = .004 and *P* = .041, respectively; Figure 5*B*).

## DISCUSSION

We have described, for the first time, the cellular response to oral vaccination at the human intestinal mucosa. We demonstrate that Ty21a vaccination generates both polyfunctional Ty21a-responsive and heterologous influenza virus–responsive T cells at the duodenal mucosa. The frequency of Ty21a-responsive and influenza virus–responsive T cells at the duodenal mucosa were robustly and consistently correlated. In contrast, cellular and humoral measurements made in peripheral blood were poorly correlated with one another and did not provide insight into the induction of heterologous responses at the duodenal mucosa.

While robust Ty21a and heterologous influenza virus–responsive CD4^+^ and CD8^+^ T cells were observed at the duodenal mucosa, no cellular response was observed at the colonic mucosa to either antigen in either T-cell subset. This suggests that duodenal responses were compartmentalized to the embryological midgut, which includes the terminal ileum, the likely site of active invasion by Ty21a [1]. Interestingly, the frequency of Ty21a-responsive CD4^+^ and CD8^+^ T cells correlated robustly with one another and with the frequency of heterologous influenza virus–responsive CD4^+^ and CD8^+^ T cells at the duodenal mucosa. This suggests that the mechanisms responsible for the generation of these mucosal responses were localized and capable of influencing T cells of different phenotypes and specificities.

Polyfunctionality among Ty21a-responsive T cells at the duodenal mucosa and in peripheral blood may point toward an important mechanism through which Ty21a confers protection against disease [20, 21]. Further, the generation of anti-LPS immunoglobulin, an accepted indication of vaccine efficacy, was closely associated with cellular responses at the duodenal mucosa, suggesting that mucosal cellular activity may be associated with the generation of protective immunity.

In murine models, influenza virus infection results in the recruitment of previously primed heterologous T cells to the lung [17]. We propose that a similar mechanism was responsible for the generation of heterologous responses at the duodenal mucosa. Although only 2 individuals had previously been vaccinated against influenza, all were likely to have been exposed to this mucosal pathogen in the community—indeed, at baseline, anti-influenza virus IgG and IgA, as well as influenza virus–responsive CD4^+^ T cells, were detected in all volunteers, not just those who had been previously vaccinated against influenza.

Trafficking to the mucosa is largely dependent upon the expression of α_4_β_7_ and its ligand, mucosal addressin cell adhesion molecule-1 (MAdCAM-1), as well as C-C chemokine receptor 9 and its ligand, chemotaxis chemokine ligand 25 [24]. We hypothesize that vaccination with Ty21a upregulated the expression of mucosal homing ligands resulting in the nonspecific migration of previously primed heterologous influenza virus–responsive T cells, carrying mucosal homing receptors, to the duodenal mucosa. This effect may or may not be restricted to influenza virus–responsive T-cell subsets, and further study is warranted.

We observed that integrin β_7_ expression intensity was generally higher among both Ty21a-responsive and influenza virus–responsive cells, compared with total cell populations, in peripheral blood and at the intestinal mucosa. Multiphasic peripheral CD8^+^ T-cell responses have previously been described and attributed to the trafficking of immune cells from peripheral blood to the mucosa [8, 9]. In keeping with this, integrin β_7_ expression among peripheral CD8^+^ T cells, particularly antigen-specific subpopulations, was more intense than that among peripheral CD4^+^ T cells. Integrin β_7_ expression among CD4^+^ T-cell subsets was highly polarized, being relatively low among peripheral subsets and high among duodenal subsets. Differential integrin β_7_ expression among antigen-specific CD4^+^ T-cell populations may ensure that some peripheral T cells persist in blood and avoid sequestration. We and others have demonstrated the presence of intracellular intravascular and bone-marrow bacteria in early, late, and recurrent invasive *Salmonella* disease [25, 26]. Thus, while T cells trafficking to the mucosa likely play a key role in the early response to invasion, CD4^+^ T-cell populations that persist in peripheral blood may help to prevent intravascular dissemination and persistence at secondary systemic sites of infection.

Prior to vaccination, all volunteers had detectable baseline levels of serum immunoglobulin specific to *S.* Typhi LPS. This may be caused by antibody binding to the core or a result of environmental exposure to nontyphoidal strains bearing the same LPS O-antigens as *S.* Typhi (O-9 and O-12); *S. enterica* serovar Enteritidis expresses both O-9 and O-12 antigens, and *S. enterica* serovar Typhimurium expresses the O-12 antigen [27]. Not all vaccinated volunteers generated peripheral humoral anti-LPS responses, which is likely a reflection of the limited efficacy of this vaccine. It was interesting that mucosal cellular responses correlated strongly with serum anti-LPS immunoglobulin responses, as humoral responses to LPS have been associated with vaccine efficacy [18]. This suggests that, in individuals in whom Ty21a vaccination is not efficacious, the failure encompasses both cellular and humoral mechanisms of defense. It has previously been demonstrated that the generation of immunoglobulin following oral vaccination is dependent upon α_4_β_7_ [28]. Thus, the close association between cellular and humoral immune responses suggests that the strength of each volunteer's cellular and humoral response to vaccination was influenced by a mucosal mechanism, possibly the expression intensity of α_4_β_7_ and/or MAdCAM-1 at the time of vaccination.

It is possible that, through Toll-like receptor engagement, vaccination may have nonspecifically activated mucosal antigen-presenting cells and T cells, resulting in enhanced cytokine production following experimental restimulation [29, 30]. However, as background cytokine production in the negative control was minimal, and as cytokine production in the positive control was not different in the vaccinated volunteers as compared to the unvaccinated volunteers, we believe this is unlikely to be the primary factor responsible for the observed heterologous cellular response. Owing to the invasive nature of endoscopic biopsy, we were limited to sampling from intestinal sites at a single time point. Data presented here and elsewhere [8] indicate that the assessment of mucosal immunity at alternate time points could provide further insight into the generation of protective immune responses. We assessed the expression of just 3 cytokines and, as a result, the T-cell populations identified here are unlikely to represent the responsive populations in their entirety.

In future studies, we plan to study the effect of oral vaccination with Ty21a on the cellular response to a range of heterologous antigens. We will comprehensively assess cellular phenotype and functionality through the inclusion of additional surface markers and a broader repertoire of cytokines/chemokines. We also plan to further investigate the timing and longevity of peripheral and mucosal immune responses.

Taken together, our data demonstrate that oral vaccination with Ty21a generates Ty21a-responsive T cells as well as heterologous influenza virus–responsive T cells at the duodenal mucosa. We propose that heterologous influenza virus–responsive T cells previously primed through natural exposure were recruited to the duodenal mucosa through compartmentalized upregulation of homing ligands. The direct evaluation of mucosal cellular immune defense provides the opportunity to identify functional correlates of protection and may offer new insights into mechanisms that may be manipulated to improve oral vaccine immunogenicity, either through the development of oral adjuvants or the development of multivalent *Salmonella*-based vectors.

## Supplementary Data

Supplementary materials are available at http://jid.oxfordjournals.org. Consisting of data provided by the author to benefit the reader, the posted materials are not copyedited and are the sole responsibility of the author, so questions or comments should be addressed to the author.

Supplementary Data
