# Supplementary material for: Oral Typhoid Vaccination With Live-Attenuated Salmonella Typhi Strain Ty21a Generates Ty21a-Responsive and Heterologous Influenza Virus–Responsive CD4+ and CD8+ T Cells at the Human Intestinal Mucosa
Source: J Infect Dis. 2016 Jan 24;213(11):1809–19. doi: 10.1093/infdis/jiw030 (PMC4857474; doi:10.1093/infdis/jiw030)
Supplement: Supplementary Data [file supp_213_11_1809__index.html]

Oral Typhoid Vaccination With Live-Attenuated Salmonella Typhi Strain Ty21a Generates Ty21a-Responsive and Heterologous Influenza Virus–Responsive CD4+ and CD8+ T Cells at the Human Intestinal Mucosa — Supplementary Data 

# Oral Typhoid Vaccination With Live-Attenuated *Salmonella* Typhi Strain Ty21a Generates Ty21a-Responsive and Heterologous Influenza Virus–Responsive CD4+ and CD8+ T Cells at the Human Intestinal Mucosa

## Supplementary Data

Supplementary Data

- Supplementary Data - docx file
