# Supplementary material for: Oral Typhoid Vaccination With Live-Attenuated Salmonella Typhi Strain Ty21a Generates Ty21a-Responsive and Heterologous Influenza Virus–Responsive CD4+ and CD8+ T Cells at the Human Intestinal Mucosa
Source: J Infect Dis. 2016 Jan 24;213(11):1809–19. doi: 10.1093/infdis/jiw030 (PMC4857474; doi:10.1093/infdis/jiw030)
Supplement: Supplementary Data [file supp_jiw030_jiw030supp_data.docx]

**SUPPLEMENTARY MATERIALS AND METHODS**

**Mucosal mononuclear cell (MMC) isolation**

Biopsies were collected on ice in R15 medium (RPMI-1640 supplemented with 15% foetal bovine serum (FBS), 2% 200mM L-glutamine and antibiotic/antimiotic). Biopsies were incubated in CII-S medium (R15 medium supplemented with collagenase II-S (Sigma-Aldrich) (0.5mg/mL)) at 37°C for 30 minutes at a 45° angle with shaking (220rpm). Mucosal biopsies were mechanically disrupted by passing the tissue suspension through a 16-gauge blunt-ended needle five times. The suspension was then passed through a 70µm cell strainer and the cell suspension stored on ice. Tissue fragments captured in the cell strainer were transferred back into the original tube by rinsing the cell strainer with CII-S medium. The process of incubation, disruption and filtration was then repeated twice more. Cell suspensions were pooled, washed, counted and seeded in R20 medium (RPMI-1640 supplemented with 20% FBS, 2% 200mM L-glutamine and antibiotic/antimiotic) in 24-well flat bottomed plates at a concentration of 2 × 10^6^ cells/well. Cells were then rested overnight at 37°C in 5% CO_2_. The following day, cells were harvested, recounted, and seeded in complete medium (RPMI-1640 supplemented with 10% FBS and 2% 200mM L-glutamine) in 96-well flat-bottomed plates at a concentration no less than 5 × 10^6^ cells/well.

**Peripheral blood mononuclear cell (PBMC) isolation**

Peripheral blood samples were diluted with an equal volume of Dulbecco's phosphate-buffered saline (Dulbecco’s PBS) (Invitrogen) and PBMCs isolated by differential centrifugation using Lymphoprep™ (Axis-Shield). Cells were washed twice in Dulbecco’s PBS, counted and seeded in complete medium in 96-well flat-bottomed plates at a concentration of 1 × 10^6^ cells/well.

**Flow cytometric analyses**

Following incubation, PBMCs and MMCs were washed with RPMI-1640 and stained for viability (Vivid®; Invitrogen) and surface phenotype using antibodies specific to CD3-APC (HIT3a; BD Biosciences), CD8-PE-Cy7 (RPA-T8s; BD Biosciences), CD4-APC-H7 (SK3; BD Biosciences), integrin β_7_-PE-Cy5 (FIB504; BD Biosciences), CD14-Pacific Blue (M5E2; BD Biosciences) and CD19-Pacific Blue (HIB19; BD Biosciences). Following fixation and permeabilisation (Cytofix/Cytoperm™; BD Biosciences), cells were stained for intracellular interferon (IFN)γ-AF700 (B27; BD Biosciences), tumour necrosis factor (TNF)α-AF488 (MAb11; BD Biosciences) and interleukin (IL)2-PE (MQ1-17H12; BD Biosciences). Cells were washed in Perm/Wash™ (BD Biosciences), resuspended in CellFIX™ (BD Biosciences), and stored in the absence of light at 4°C until data were acquired using a LSR II flow cytometer (BD Biosciences).

Compensation beads (BD Biosciences) were used to create compensation matrices and sequential cell isolation used to identify populations of interest (Figure 2). IFNγ^+^, TNFα^+^ and IL2^+^ populations were positively identified and combinatorial expression profiles determined using FlowJo version 7.6.5 (Treestar Inc.). Geometric mean integrin β_7_ expression intensity was assessed in total CD4^+^ and CD8^+^ lymphocyte populations. Geometric mean integrin β_7_ expression among cytokine-producing subpopulations (IFNγ^+^, TNFα^+^ and IL2^+^) was assessed using the geometric mean of the three cytokine-producing populations.

**SUPPLEMENTARY FIGURE LEGENDS**

**
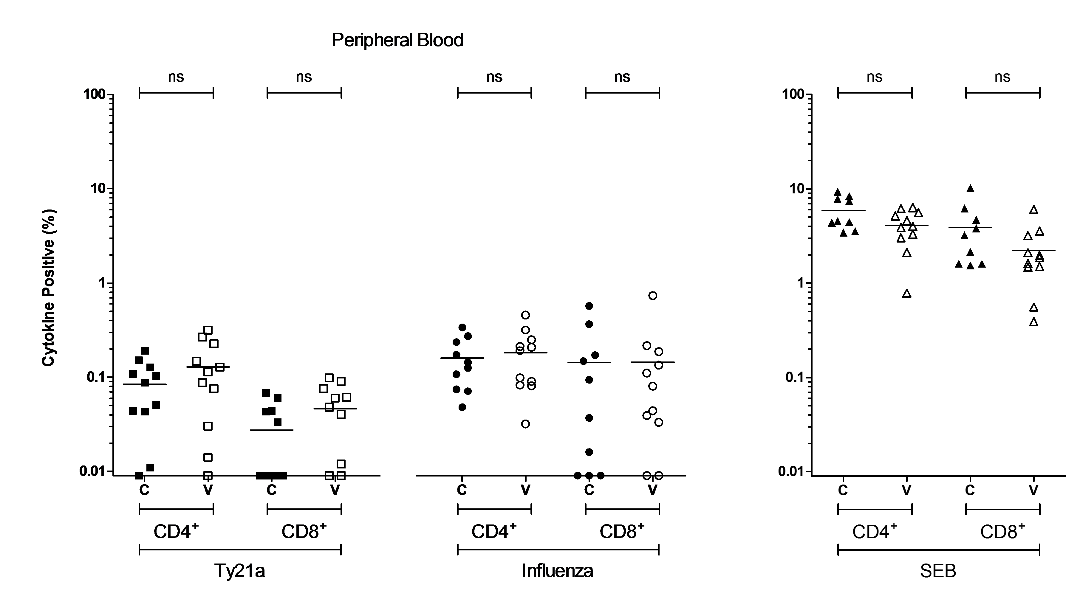
**

**FIGURE S1. Antigen-specific cytokine-producing populations at day 0.** The frequency of CD4^+^ and CD8^+^ Ty21a-responsive and heterologous influenza-responsive populations expressing any combination of IFNγ ± TNFα ± IL2. SEB-stimulated control data is also included. For control (C; closed squares, circles and triangles) and vaccinated (V; open squares, circles and triangles) volunteers, measurements were made in peripheral blood. Horizontal bars represent mean values (comparisons were made using unpaired *t* tests).

**
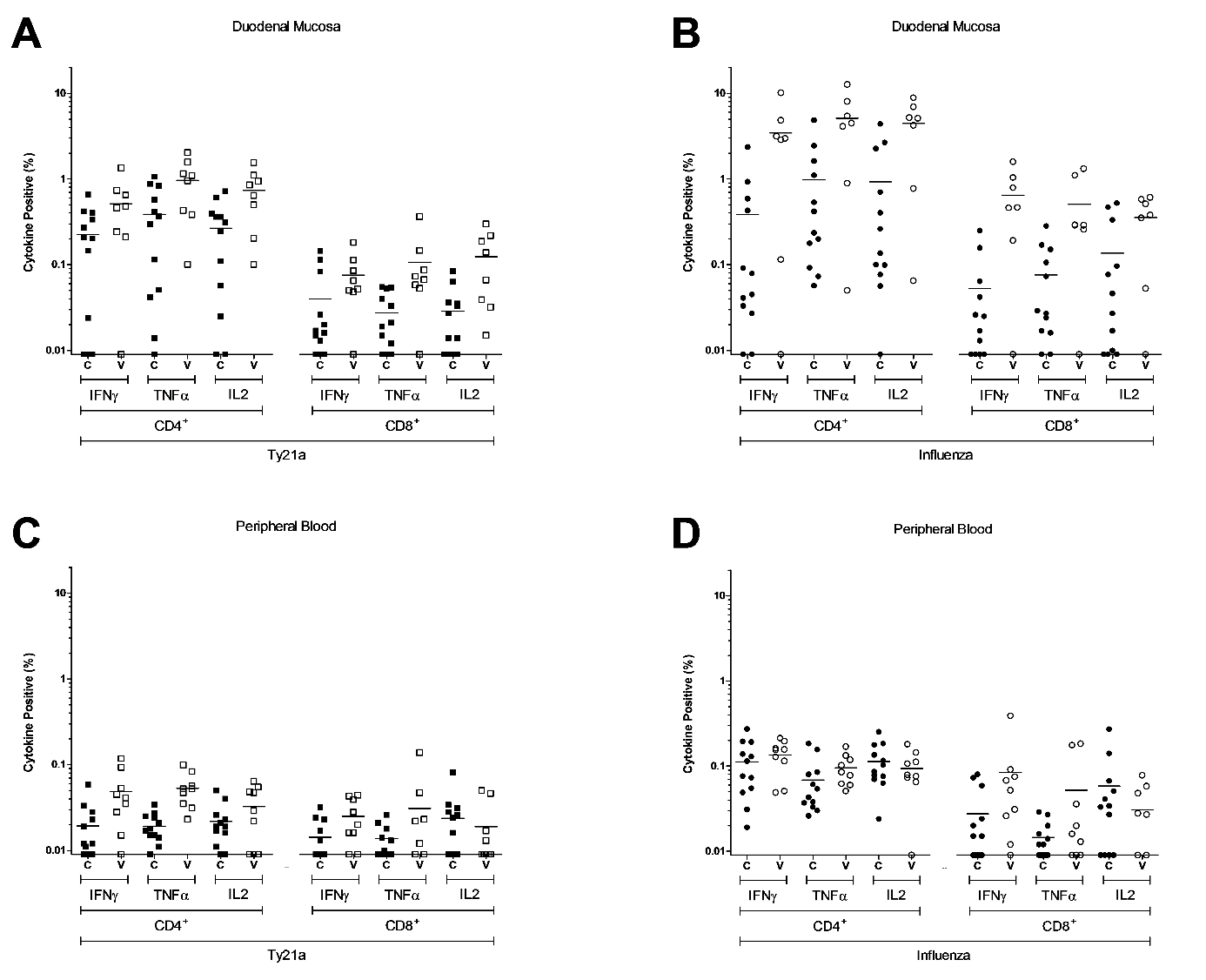
**

**FIGURE S2. Antigen-specific production of individual cytokines at day 18.** The frequency of CD4^+^ and CD8^+^ Ty21a-responsive and heterologous influenza-responsive populations expressing IFNγ, TNFα, or IL2 above background. For control (C; closed squares, circles and triangles) and vaccinated (V; open squares, circles and triangles) volunteers, measurements were made at the duodenal mucosa (**A and B**), and in peripheral blood (**C and D**). Horizontal bars represent mean values.
